# John squire and endothelial glycocalyx structure: an unfinished story

**DOI:** 10.1007/s10974-022-09629-x

**Published:** 2022-10-19

**Authors:** Kenton P. Arkill, C. Charles Michel, Elizabeth V. M. Rider, Elise A. Wood, Mathew O. Small, Jennifer L. E. Brown, Abigail L. Kinnaird

**Affiliations:** 1 School of Medicine, University of Nottingham Biodiscovery Institute, Nottingham, UK; 2 Department of Bioengineering, Imperial College London, London, UK

**Keywords:** Vascular permeability, Reflection coefficient, Albumin, Proteoglycan, Glycosaminoglycan

## Abstract

John Squire did not only produce leading works in the muscle field, he also significantly contributed to the vascular permeability field by ultrastructural analysis of the endothelial glycocalyx. Presented here is a review of his involvement in the field by his main collaborator C.C. Michel and his last postdoctoral researcher KP Arkill. We end on a reinterpretation of his work that arguably links to our current understanding of endothelial glycocalyx structure and composition predicting 6 glycosaminoglycans fibres per syndecan core protein, only achieved in the endothelium by dimerization.

**Supplementary Information:**

The online version contains supplementary material available at 10.1007/s10974-022-09629-x.

## How john squire came to work on the structure of the endothelial glycocalyx

It is in the microcirculation (capillaries, smallest arterioles and venules) that oxygen and nutrients are delivered from blood to tissues and carbon dioxide and other waste products of metabolism removed to be carried to the organs of excretion. Only the tiniest fraction of the fluid coursing through the microvessels is lost from the plasma while this occurs, in spite of the large hydrostatic pressure difference between the plasma and the tissues. At the end of the nineteenth century, it was recognized that the walls of the capillaries and smallest venules acted as ultrafilters, which are a barrier to plasma macromolecules but through which water and small hydrophilic molecules can diffuse freely. This leads to sustained differences in osmotic pressure across microvascular walls resulting from the differences in protein concentrations between the plasma and the interstitial fluid (ISF). First identified by Starling (Starling 1896) it slowly came to be accepted that this osmotic pressure opposed the greater hydrostatic pressure in the microcirculation, which is necessary to maintain microvascular blood flow. The osmotic pressure of the plasma proteins (or colloidal osmotic pressure, COP) was found to have similar values to indirect estimates of average microvascular pressures. Acting as if to draw fluid from ISF to plasma, it holds fluid in the circulation while permitting the rapid diffusional exchange of small solutes such as glucose, amino acids and urea to occur between plasma and ISF. Ingenious experiments deduced that the ultrafilter of microvessels in mammalian skeletal muscle acted as if it were an impermeable membrane penetrated by cylindrical pores with radii in the range of 3.5–4 nm, just a little greater than the Stokes–Einstein radius of the serum albumin molecule (Pappenheimer et al. 1951; Grotte 1956).

Not until after developments in biological electron microscopy in the mid twentieth century, was it possible to discover the nature of this ultrafilter. Early electron micrographs revealed that endothelial cells in many tissues contained numerous vesicles, which could be labelled with tracers added to the circulation. Throughout the 1960’s to the mid-1990’s, much attention was focused on the vesicles as transporters of plasma macromolecules (Bruns and Palade 1968; Bruns and Palade 1968) and on the inter-cellular pathway between endothelial cells with the tight regions of the intercellular junctions acting as the ultrafilter (Karnovsky 1967). A small minority of investigators; however, were unsatisfied with the experimental evidence and inconsistencies in the theory of flow through the tight junctions and turned their attention to the endothelial glycocalyx as a possible candidate for the ultrafilter in microvascular walls.

The endothelial glycocalyx is a layer of glycosaminoglycans and proteoglycans on the luminal surface of the endothelial cell lining blood vessels. Although its possible presence had been suggested in the days before electron microscopy of the vasculature (Chambers and Zweifach 1947), the early electron micrographs of microvessels provided no evidence for its existence. Its presence was first established by Luft (Luft 1966) who stained thin sections of tissues using ruthenium red. Ruthenium red revealed a heavily stained coat on the luminal surface of the endothelial cells which extended into the intercellular clefts, covered the fenestrations of fenestrated endothelia, and lined the endoplasmic vesicles and caveolae. Perfusion of capillaries with solutions containing native ferritin molecules revealed a much lower concentration of ferritin in the vesicles and caveolae, and this was consistent with their exclusion from the ruthenium red staining layer (Loudon, Michel et al. 1979; Clough and Michel 1981). Using these and other data, Curry & Michel (Curry and Michel 1980) wrote a short paper proposing the glycocalyx as the microvascular ultrafilter and putting together quantitative expressions for its hydraulic conductivity (permeability), its partition coefficient for solutions of water soluble solutes of different molecular radii, its diffusional permeability to these solutes and its reflection coefficients. Here they were guided by expressions derived by Ogston and his colleagues (Ogston 1958, Ogston, Preston et al. 1973) for the partition and diffusion coefficients of molecules in polymeric gels. These were derived for the distribution and diffusion of spherical solute molecules within and through a random arrangement of long cylindrical molecules of the gel and were found to describe distribution and diffusion of molecules through hyaluoran gels in vitro. The same expressions however, predicted larger values of the partition coefficients and hence smaller values for reflection coefficients of serum albumin than those estimated for microvascular walls. By the time their paper was published both Curry and Michel had independently realized this inconsistency could be resolved if the long cylindrical molecules of the gel were regularly rather than randomly spaced (Michel 1981).

In the summer of 1984, Michel, discussed this hypothesis, in a lecture he gave at Imperial College London. At the end of the lecture, the chairman, Professor David Blow, who was Head of the Biophysics at Imperial, suggested that Michel should talk to a member of his department, John Squire, who had developed methods for detecting evidence for underlying ordered structures in electron micrographs. Squire was not immediately available but Michel remembered his name. It was over two years later before they met, by which time, Michel had moved to work in London. After their first meeting, Squire was immediately interested in the project and agreed to collaborate. In their first project together, they examined the glycocalyx of cultured endothelial cells prepared by Dr Una Ryan. The results were promising and presented as a demonstration to the Physiological Society in 1989 (Luther et al. 1989).

## Initial studies

Experience from examining the glycocalyx in cultured endothelial cells gave confidence to apply for a project grant from the Wellcome Trust. From the observations on cultured endothelium, it was clear that the glycocalyx was not a regular lattice of proteoglycans which might be analysed using crystallographic image-processing methods. Squire decided to use self- or autocorrelation functions (ACF) on the micrographs, which is unconventional compared to quantifying Fourier transforms directly. Using the ACF analysis, Squire and his colleagues had found they were able to detect underlying periodicity in the density of electron micrographs of apparently disordered structures (Squire 1972). We believed we should use this approach to examine the glycocalyx of microvessels which had been rapidly frozen from the living state without chemical fixation, then fractured, deep-etched by submimation, and uni-directionally coated (shadowed) with platinum and carbon to reveal its underlying structure. Any evidence for regular periodicity from this low throughput method might then be found in electron micrographs of frog glycocalyx prepared using conventional methods. In writing the application, John Squire revealed his remarkable ability to explain a complicated idea with the help of a simple diagram and a few words. He made the application into a convincing story and it was fully funded. For several reasons, we carried out the experiments on frog mesenteric capillaries. First, frog mesentery is very thin, often less than 10 µm, so that the capillaries which have diameters in the range of 15–20 µm bulge above the mesentery surface with a combined layer of mesothelium and endothelium of less than 1.0 µm between the surface and the glycocalyx. This minimized the thickness of tissue between the glycocalyx and the surface which had to be frozen rapidly enough for satisfactorily freezing of the glycocalyx. Further reasons for working on frog capillaries were that they had been the subject of many investigations demonstrating the ultrafiltration properties of microvascular walls and showing the exclusion of native ferritin molecules from the caveolae and endoplasmic vesicles.

**Fig. 1:**
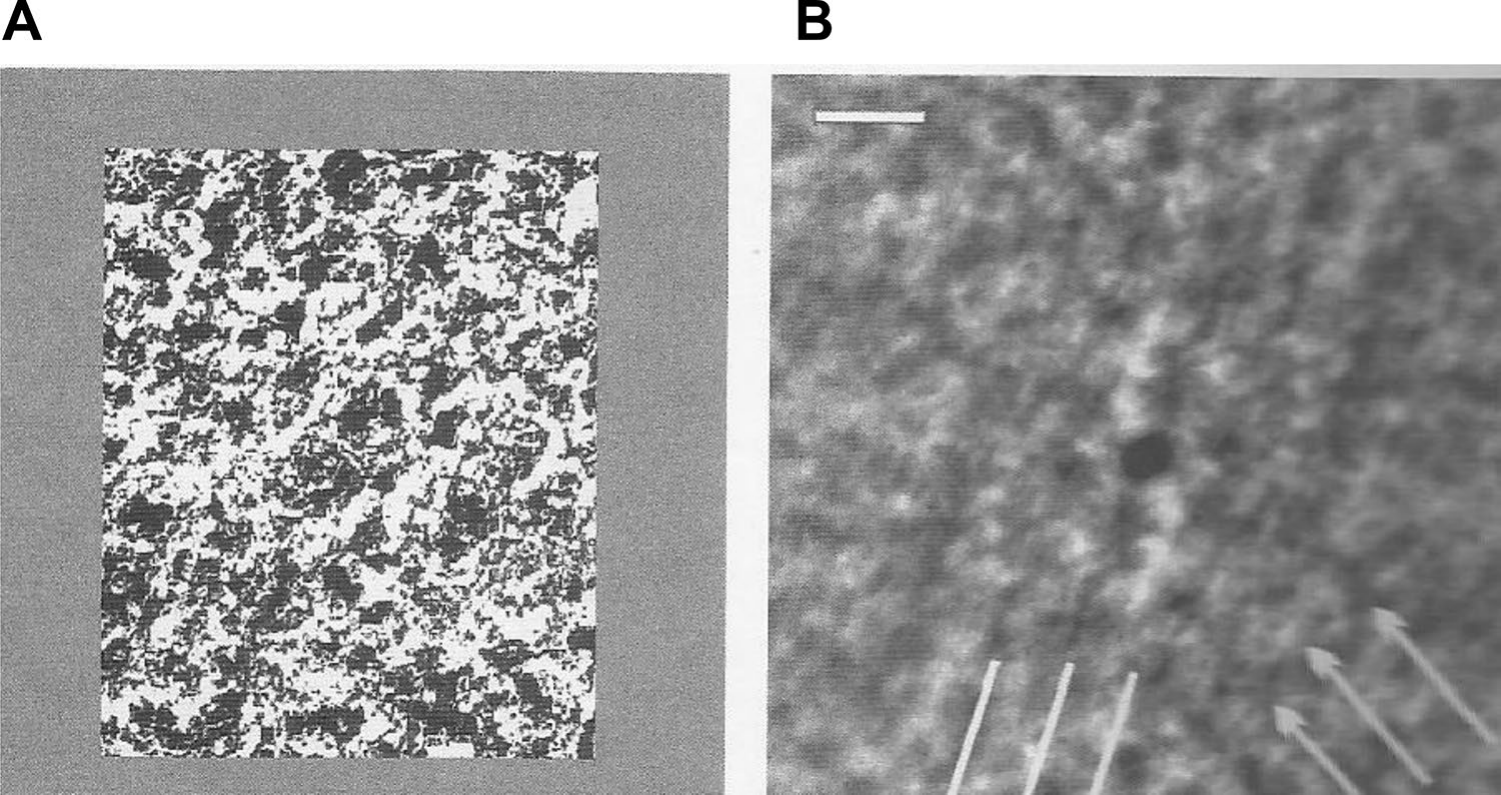
Freeze fracture measurement on the endothelial glycocalyx of a frog mesenteric capillary Taken from (Squire et al. 2001). **A** Micrograph from the technically challenging (including due to determining regions of interest, depth of freezing) untreated slam frozen and freeze fractured. **B** The auto-correlation of the (**A**) which reveals a striking quasi-hexagonal arrangement within the glycocalyx with spacing of between 80 and 120 nm as indicated by the white lines and arrows. Smaller periodicities can also be found within these major repeats. White scale bar is 200 nm

In spite of the advantages of frog mesenteric vessels being the subjects of choice, success in achieving satisfactorily frozen tissue, which had been in healthy state up to the instant of being frozen, proved to be even more difficult than had been anticipated. This was in spite of using the Heuser slam freezing technique (Heuser 1981; Hirokawa and Heuser 1981). To be sure that the tissue remained in a healthy state up to the instant of being frozen, its exposed surface had to be kept moist with frog Ringer solution. It was essential to keep this surface liquid as thin as possible, a condition often thwarted by menisci of Ringer solution around the bulging profiles of the vessels on the exposed mesenteric surface. Even when a satisfactory freeze had been obtained, it was then a matter of chance whether the plane of the fracture through the preparation managed to sample the glycocalyx in a useful way. Fortunately, after many failures, Michael Chew was able obtain some beautiful preparations (Fig. 1).

Electron micrographs of these, enabled this project to become part of a five-year programme grant. Further success was achieved with rapidly frozen, lightly glutaraldehyde fixed, specimens of the frog lung capillaries, where the fracture plane passed through the glycocalyx roughly parallel to its surface. Analysis of successful freeze-fracture preparations using the ACF revealed a regular spacing parallel to the endothelial surface of approximately 20 nm (mean 19.6 nm, SD = 3, n = 9) and a similar regularity in about half the specimens examined perpendicular to the cell surface (mean 22.6 nm, SD = 3.6 nm, n = 5). There was also evidence for a large, roughly hexagonal lattice with spacing between 80 and 120 nm. Squire et al. made a strong case for believing the pillars of the ~ 20 nm lattice were cylindrical structures with diameters between 10 and 16 nm and as such could represent the ultrafilter of microvascular walls with open channels between them excluding spherical molecules of radii greater 4 nm (Squire et al. 2001). The larger spacing might suggest points in the glycocalyx which had transmembrane links. Satcher et al. (Satcher et al. 1997) had reported that in cultured bovine endothelial cells, rapid freeze, freeze-drying and metal coating treatment of the sub-membrane cytoskeleton formed a square lattice with unit length of ~ 100 nm. If the sub-membrane cytoskeleton formed a similar structure in endothelia in vivo, the corners of this lattice could be the sites of links between the glycocalyx and the cell interior.

Having gained some insight into the structure of glycocalyx in the absence of chemical fixation, we now turned to the question of whether these characteristics were to be found in sections of tissue prepared using more conventional methods of electron microscopy with chemical fixation and either staining or labelling of the glycocalyx. In some vessels the glycocalyx was stained with ruthenium red, in others with alcian blue. We also used electron micrographs from earlier studies where the glycocalyx had been labelled using a highly cationised form of ferritin (Clough 1982; Turner et al. 1983) ACF’s of the densities of the glycocalyx, stained with ruthenium red or Alcian blue, running parallel to the endothelial cell surface revealed a periodicity of ≈ 20 nm consistent with the spacing of density in the freeze-fracture preparations. Similar values were measured on ferritin labelled glycocalyx.

It seemed that the periodicity of density revealed by the ACF, indicated a regularity in glycocalyx structure. In attempting to model how this might be represented, we were strongly influenced by a paper of Rostgaard & Qvortrup (Rostgaard and Qvortrup 1997). Using a novel fixation method, these authors had examined the glycocalyx covering the fenestrated endothelium of microvessels in the intestinal villi, gastric mucosa, and the peritubular and glomerular capillaries of the kidneys of Wistar rats. In all these endothelia, the fenestrae were 60–70 nm in diameter and were covered by a thin (≤ 5 nm) diaphragm. On the luminal, but not the abluminal side of the fenestrae, was a multi-filamented plug. Each plug was made up of 20–40 brush-like filaments each 5–10 nm thick, extending up to 400 nm towards the lumen of the vessel. Rostgaard and Qvortrup [23] suggested the spacing between the filaments might be in the range of 10 nm and 2–3 nm between the proteoglycan brush like fibres.

This led Squire to suggest the model based on quantification of ACF of the glycocalyx of frog microvessels. He suggested that the core proteins of the glycoproteins were rooted in the cell membrane at points forming a square lattice of unit length 100 nm and perhaps communicated with the submembrane cytoskeleton. On the luminal side of the membrane, the core protein sprouts the tufts of glycoprotein and suggests that these are set 20 nm apart.

The great advantage of using the ACF was that it removed subjective influence in the interpretation electron micrographs. We agreed that it would excellent if we could analyse Rostgaard and Qvortrup’s micrographs. In 1999, with this in mind, Michel wrote to Professor Rostgaard suggesting a possible collaboration. After some months Michel received a letter from Dr Qvortrup, apologizing for the delay but explaining that Professor Rostgaard had suffered a severe stroke and had not been able to reply to Michel’s inquiry. Qvortrup was enthusiastic about a collaboration and believed that Rostgaard would be also. He offered to send us both original micrographs and, also if necessary, the blocks from which the sections portrayed in the micrographs had been cut. Owing to the retirement of Michel and later Squire, with his subsequent move to Bristol University, it was some time before the collaboration was able to start.

## Latter and ongoing studies

Kenton Arkill started on the project in 2009, the interview was relaxed but the pub was only attended once he had accepted the role. The work was based in Dave Bates’ lab at Bristol as the funding awardees were retired (Charles Michel; John Squire), about to retire (Chris Neal), or not in Bristol (Carlo Knupp). Fortunately, Klaus Qvortrup could visit and help interpret the Danish handwritten notes for the hundreds of negatives taken over > 10 years, this was also aided by the TEMs rotating the image at different magnifications to ensure we knew the scales were correct. We essentially confirmed the frog findings on rats and rabbits (19.5 nm core spacing) on the Rostgaard & Qvortrup samples (fluorocarbon-tannic acid stained) although the longer spacings were inconsistent (Arkill et al. 2011). This 2D work highlighted two issues, firstly most of the stains act as a mordent and clump the endothelial glycocalyx, and secondly the transmission electron microscopy techniques are normally looking across the vascular wall, not from the lumen to the wall as the transported molecules would be travelling. We attempted to address this using tilt series tomography and lanthanide staining, the latter dubbed lanthanum dysprosium glycosaminoglycan adhesion (LaDy GAGa) technique (Arkill et al. 2012, Arkill, Qvortrup et al. 2014). Even in the low number of 3D samples there was still a 20 nm spacing. The LaDy GAGa method seems to give good fibres but tends to be sheared off easily, which whilst adequate to determine ultrastructure, is a major limitation to any systematic approach needed to determine pathological structural changes in the glycocalyx. There has always been an issue with the spacing work, which has been alluded to here, the glycosaminoglycan fibres are clumped around the core protein. So, whilst the values work, in physiology the filtration is unlikely to be between core proteins with a clump of glycosaminoglycans around them as the GAGs are likely to be unraveled and stretching into the lumen.

**Fig. 2:**
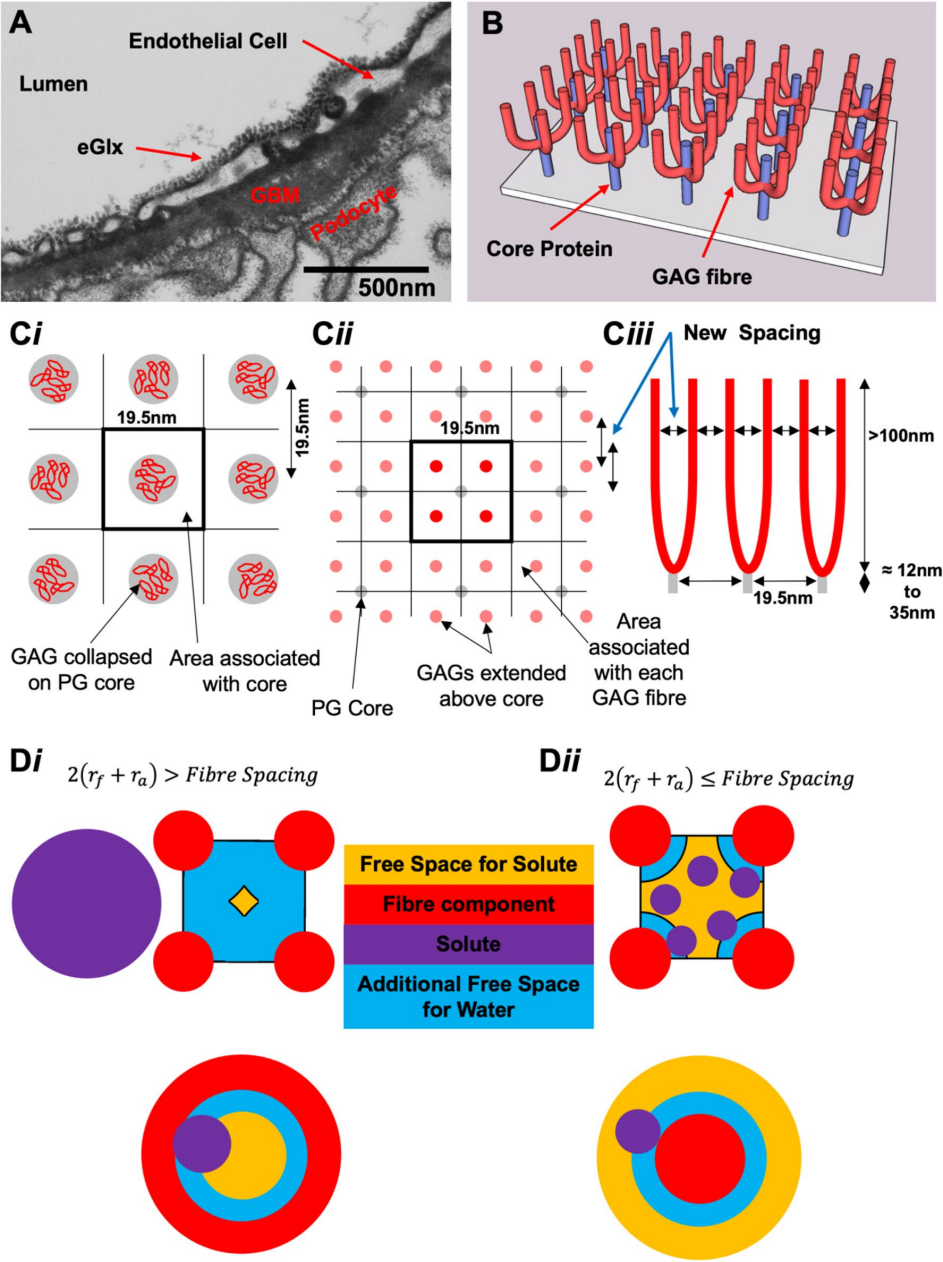
Relating the fibre spacing to the reflection coefficient. **A** From(Arkill et al. 2011) Rostgard-Qvortrup staining of the endothelial glycocalyx (eGlx) in the rat kidney glomerulus showing short clumps. **B** Proteoglycan cores (blue) with example of glycoasaminoglycan (GAG) fibres attached mid rotation to plan view (See Supplemental video 1). **C**i The Squire filtration model (exampled on a square lattice for ease of display) (Squire et al. 2001; Arkill et al. 2011). The GAG fibres (red) are collapsed on the core, which can be expanded (**C**ii). **C**iii represents the new expanded model in cross-section where there is a new inter-fibre spacing. **D** shows how the free space for a solute to travel is dependent on the relative molecular size (${r}_{f}$= fibre radius; ${r}_{a}$= solute radius). **D**i The solute cannot fit freely around the fibres so can be approximated to a tube. The reflection coefficient can be determined via Anderson and Malone’s calculations (Anderson and Malone 1974). **D**ii If the solute can fit around the fibres the available space can be approximated to an annulus and the reflection coefficient can be determined via Zhang et al.’s calculations (Zhang et al. 2006). **C**i–iii from and (**D**) adapted from (Arkill 2021)

Upon John Squire’s passing, Kenton Arkill tested the hypothesis that the 20 nm spacing was indeed from the core proteins but was not the filtration component itself (Fig. 2). The filtration would come from the unraveled GAG chains. If one unravels the multiple chains there would be a smaller spacing, but of course the individual chains are much thinner leaving the pore for molecular transport a similar size. When our best estimate for the parameters are put to this thought experiment we find that one would need a mean of ≈ 6 GAG chains to each core, which is consistent with endothelial syndecans in dimers, which in turn is the most likely physiological confirmation (Godmann et al. 2020). We can therefore conclude that John Squire’s team’s structural work does, or at very least can, now reflect the biochemistry we expect in physiology.

**Fig. 3:**
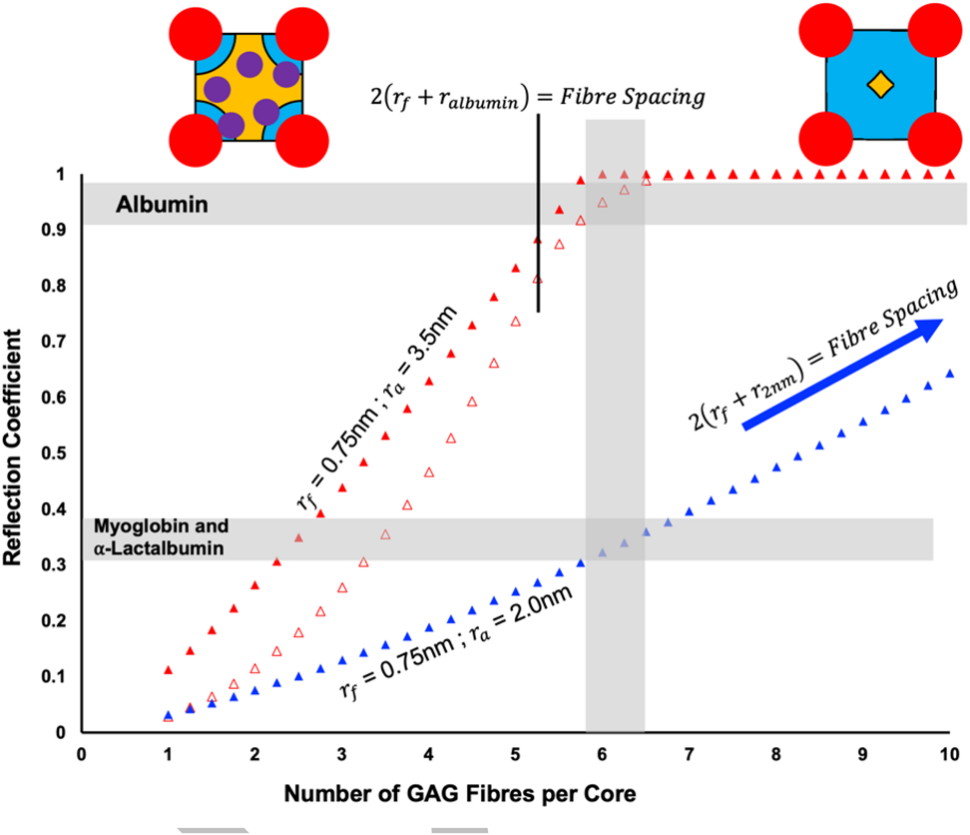
The unraveled glycosaminoglycan (GAG) model predicts a mean of ≈ 6 fibres per proteoglycan core. The graph depicts the model for a spacing of 19.5 nm between proteoglycan cores in a hexagonal arrangement of cores and unraveled glycosaminoglycans with a fibre radius (${r}_{f}$) of 0.75 nm. Parameter justification for albumin (${r}_{3.5nm}$) using the tubular model (open red triangles) are in (Arkill 2021). The red filled triangles are using the annular Zhang model (Zhang et al. 2006) to determine the reflection coefficients, these are always higher than the tubular model for the same number of fibres per core and less applicable to albumin. The blue filled triangles are the annular Zhang model for ${r}_{2nm}$ to approximate myoglobin and ⍺-lactalbumin

Using a solute’s available space compared to water to determine by calculation value for the solute’s reflection coefficient. This calculation has several assumptions including long fibers and limited solute interaction; further the shape of the pore can significantly change the outcome. Arkill used the tube model from Anderson and Malone (Anderson and Malone 1974) and this seems reasonable for albumin with its large 7 nm diameter (Fig. 2Di). However, for smaller solutes such as myoglobin [$r$=1.9 nm (La Verde et al. 2017)] or $\alpha$-lactalbumin [$r$≈2.0 nm (Gast, Zirwer et al. 1998)] where there have also been some experimental determinations of their reflection coefficients perhaps a better model would be that of Zhang et al. (Zhang et al. 2006) where there is an annulus made of an excluding fibre and the space around it (Fig. 2Dii). Figure 3 shows the difference in calculated reflection coefficient for the two models as the mean number of GAG fibres/core is increased. Depicted here are values for ${r}_{f}$ = 0.75 nm justified for negatively charged molecules previously (Arkill 2021). Experimentally myoglobin and ⍺-lactalbumin have reflection coefficients of 0.35–0.38 (Michel 1980; Curry et al. 1987) and 0.34 (Huxley et al. 1993) respectively. The unravelled Squire model works for 6–6.5 GAG fibres per core.

There are of course several important caveats to these conclusions including myoglobin’s surface charge in terrestrial animals is only slightly negative at physiological pH (Mirceta et al. 2013), which would reduce the effective ${r}_{f}$. Further there are higher reflection coefficient measurements (0.57) by a slightly different method (Rippe and Haraldsson 1986). Perhaps the biggest question is how the hyaluronan, with a less negative fixed charge density, fits into the endothelial glycocalyx as this would alter the models that are based on micrographs from charge-based staining. The other prediction from Squire et al. (Squire et al. 2001) is on the endothelial glycocalyx being bound to the actin cytoskeleton. This is based on the 100 nm longer spacings from the freeze fracture experiments and the similarity to the Satcher et al. (Satcher et al. 1997) work using a similar freezing method. Whilst there is some doubt over this freezing method’s ability to retain the actin ultrastructure (Small et al. 1999), it is conclusive from biomolecular studies that the transmembrane syndecans can transiently bind to the actin cytoskeleton (Yoneda and Couchman 2003; Multhaupt et al. 2009; Li and Wang 2019). The question of if this binding changes the ordering of the proteoglycan cores, and the solute reflection coefficient accordingly, is as yet unanswered.

John Squire was an excellent scientist who led by great moral example. His work is seminal in this field, and methods are still used. He certainly influenced me (KPA), both because my scientific career is an extension of his work, but also as an inspiration of how to put people and science values first.

### Supplementary Information

Below is the link to the electronic supplementary material.

Supplementary file1 (MP4 14887 KB)
